# Inter-implant distance and buccal bone thickness for a novel implant design: a preclinical study

**DOI:** 10.1007/s00784-023-04942-2

**Published:** 2023-03-22

**Authors:** Stephen Chen, Ivan Darby

**Affiliations:** Periodontics, Melbourne Dental School, The University of Melbourne, Victoria, Australia

**Keywords:** Swine, Miniature, Dental arch, Mandible, Dental implantation, Endosseous, Alveolar process, Bone remodelling

## Abstract

**Objectives:**

This study assessed bone height between novel tapered implants at different inter-implant thread peak (TP) distances, and the impact of TP distance from outer buccal bone (BB) on marginal bone levels (MBL).

**Materials and Methods:**

Fully tapered implants with 0.5-mm thread depth and TP diameter 1 mm wider than the shoulder diameter were placed in healed ridges of minipigs. On one side, four implants were placed with inter-implant TP distances of 1, 2, or 3 mm corresponding to inter-implant implant shoulder distances of 2, 3, and 4 mm respectively. Three implants were placed on the other side with TP distances to outer BB of > 1 mm, 0.5–1 mm, or < 0.5 mm. After 12 weeks, (a) first bone-to-implant contact (fBIC), total BIC, bone area-to-total area (BATA), and coronal bone height between implants (Bi ½ max) for inter-implant distance, and (b) fBIC, BIC, and perpendicular crest to implant shoulder (pCIS) for BB were evaluated.

**Results:**

No significant differences in bone healing and inter-implant bone height were noted for any of the TP distances. BB resorption was significant when TP distance to outer BB was < 0.5 mm. However, fBIC was lowest with TP to outer BB of 1.75 mm.

**Conclusions:**

Inter-implant bone height between adjacent implants can be maintained even at an inter-implant TP distance as low as 1 mm. A minimum TP to outer BB distance of 0.75 mm is required for predictable maintenance of MBL.

**Clinical relevance:**

Inter-implant distance and BB thickness are clinically relevant and require preclinical research to clarify concepts.

## Introduction

Dental implants are a well-established treatment modality for the rehabilitation of missing teeth. A key measure of clinical success is maintenance of bone at the shoulder of the implant [1], which may be influenced by patient, clinician and implant design factors. Patient factors include bisphosphonate treatment [1], uncontrolled diabetes mellitus [2], and smoking [3–5], as well as local factors such as infections and periodontal disease [4–7], bone volume and quality, and attached mucosa volume [4, 8]. Clinician factors include surgical technique and level of experience [5, 8]. Implant-design factors such as the implant surface material, diameter of the implant, implant-abutment connection, and microgap [4, 9–12] can influence marginal bone levels at implants.

When adjacent implants need to be placed, an additional factor to consider is the distance between the implants. When implants are placed too close together, the bone crest between the implants is reduced [13]. Recommendations currently exist on the minimum distance between implants when placed adjacent to each other, i.e., at least 3 mm between adjacent implant shoulders at the implant-abutment level [13, 14]. Furthermore, it has been suggested that implants should be placed at least 1 to 2 mm away from the outer edge of the buccal bone in order to maintain buccal bone height [15–19].

These guidelines were based on traditional implant designs, where the implant shoulder is the widest part of the implant. New implant systems have since been developed that have different designs. One such implant is a novel fully tapered implant where the diameter of the self-cutting implant threads is greater than that of the implant shoulder (BLX implant®, The Straumann Group, Basel, Switzerland) [20]. When measuring the distance between adjacent implants to be placed, the measurement is usually the distance from implant shoulder to implant shoulder, since this is the widest part of the implant for most implant systems. With the novel implant under investigation, however, the threads extend beyond the central implant diameter. So measuring from implant shoulder to implant shoulder may mean that the thread peak (TP) distance between adjacent implants is closer than the minimum recommended distance. It may be speculated that thread proximity may have an effect on the inter-implant crestal bone level when adjacent implants of this type are placed. A recent pre-clinical study using this novel implant design investigated submerged and transmucosal healing protocols. However, implants were widely spaced apart and this study could not be used to assess implant proximity [20].

A further consideration is the proximity of the implant to the outer surface of the buccal bone. It has been reported that implants placed too close to the buccal bone wall can have a detrimental effect on the buccal bone [21–23]. With the novel implant under investigation, measuring buccal bone thickness from the implant shoulder to the outer surface of the buccal bone may not be comparable to traditional implant designs, since the wider diameter threads will result in the TP being closer to the outer surface of the bone than the shoulder, and therefore closer to the outer buccal bone than the minimum distance recommended.

Currently, there are no data available on the effect of inter-implant TP distance and TP distance to outer buccal bone with this novel implant design. The purpose of this study, therefore, was to assess bone healing and implant bone height between implants of this novel design when placed at inter-implant TP distances of 3 mm, 2 mm, or 1 mm, and to also assess the impact of TP distances of > 1 mm, 0.5–1 mm, or < 0.5 mm to the outer buccal bone wall on maintenance of marginal bone levels (MBL) after 12 weeks of healing in a porcine model. The similarities of porcine bone to human bone make it suitable for investigations in bone regeneration in implant dentistry [24].

## Materials and methods

This study was performed in accordance with the Swedish Animal Protection Law (Animal Welfare Act 1988:534) and EU Directive 2010/63/EU on the protection of animals used for scientific purposes. The study was approved by the Ethical Committee of the University of Lund, Sweden (ethical approval no. M-192–14) and reported according to the ARRIVE (Animal Research Reporting of In Vivo Experiments) guidelines [25].

Ten female Göttingen minipigs (Ellegaard Göttingen minipigs A/S, Dalmose, Denmark), with a mean weight of 34.62 ± 2.61 kg and a mean age of 20.92 ± 0.79 months, were used in the study. The animals were acclimatised for a minimum of 1 week in standard pens (three or four animals per pen) under controlled environmental conditions. A standard soft food diet (# 801,586; Special Diet Services (SDS), Witham, UK) was given. Nine animals were used for data collection with one for calibration.

### Surgical procedure and terminal procedure

Tooth extraction and implant placement was performed three months apart in an animal surgery operating suite under full narcosis and aseptic conditions. The animals were fasted overnight prior to surgery to prevent vomiting. The authors placed all implants and undertook all measurements.

Dexmedetomidine (Dexdomitor, Orion Pharma Animal Health, FI-02101 Espoo, Finland; 25–35 µg/kg) and tiletamine-zolazepam (Zoletil 100 Vet, 06,511 Carros cedex, France, 50–70 mg/kg) were injected intramuscularly as a pre-medication anaesthetic. Thereafter, each animal was individually dosed with Propofol (PropoVet multidose, Orion Pharma Animal Health, FI-02101 Espoo, Finland) in the range of 40–100 mg/h to maintain anaesthesia. Pain relief was given pre-emptively and for up to 4 days post-surgically by means of Carprofen (Rimadyl Vet, Orion Pharma Animal Health, FI-02101 Espoo, Finland; 4 mg/kg, s.i.d.) in combination with buprenorphine (Vetergesic Vet, Orion Pharma Animal Health, FI-02101 Espoo, Finland; 0.03 mg/kg). Antibiotic prophylaxis was administered by means of a combination of benzylpenicillin procaine + dihydrostreptomycin (Streptocillin Vet, Boehringer Ingelheim Vetmedica, Ingelheim am Rhein, Germany) 25 mg/mg and 20 mg/kg s.i.d. i.m. During anaesthesia the animals were intubated for ventilator-assisted breathing and vital parameters (pulse oximetry, rectal temperature, blood pressure, CO_2_ levels) were continuously monitored. Intra-operatively, a local anaesthetic, Xylocaine in combination with adrenaline (20 mg/ml and 12.5 µg/ml respectively, Astra AB, Södertälje, Sweden) was administered (1.8-mL infiltrate injection Astra AB, Södertälje, Sweden; 20 mg/ml and 12.5 mg/ml) per hemi-mandible.

**Fig. 1:**
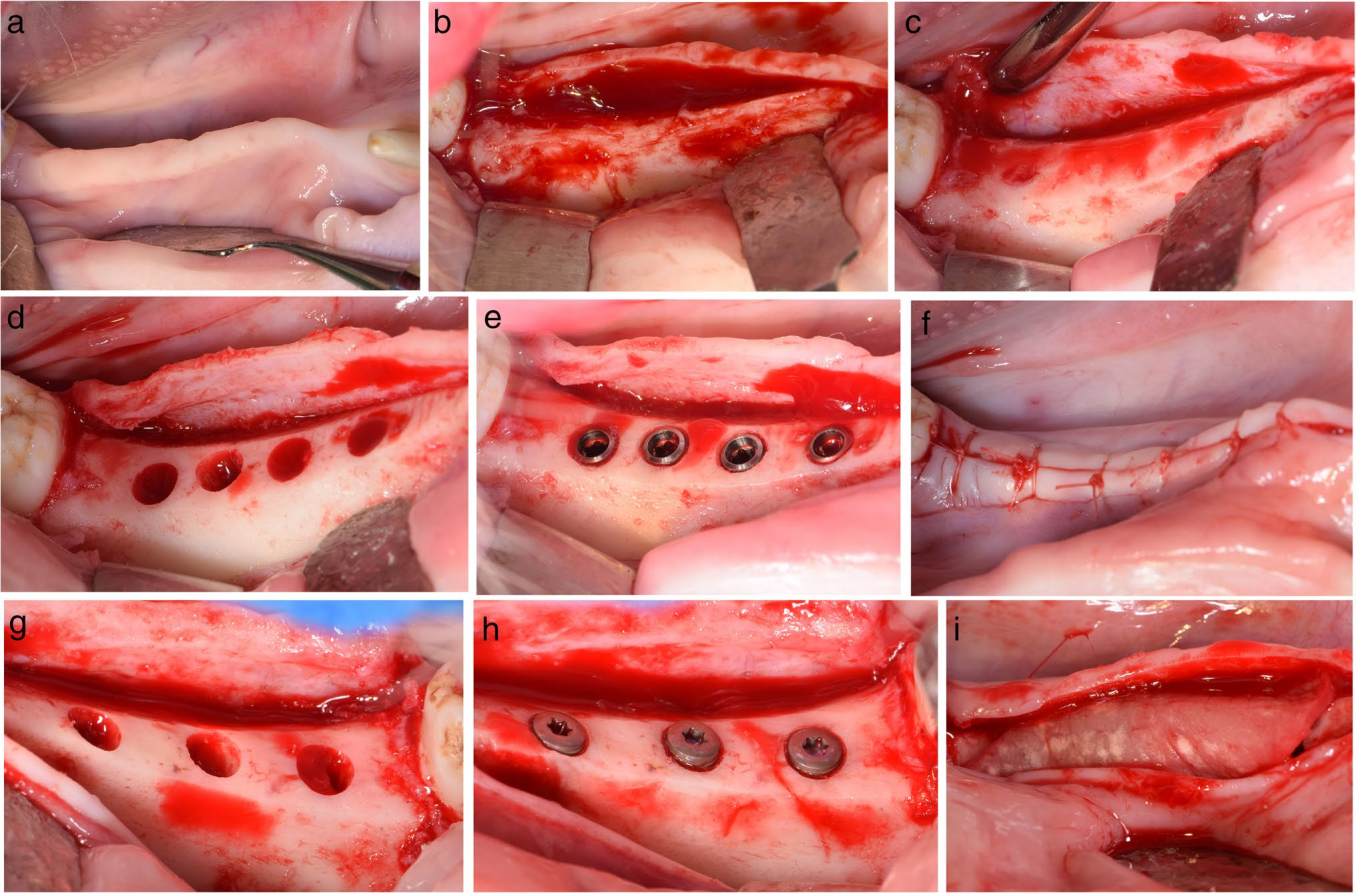
Surgical procedures. **a** Hemi-mandible 12 weeks after extraction of the mandibular premolars (P2–P4) and first molar (M1). **b** Elevation of full thickness buccal and lingual muco-periosteal flaps. **c** Gentle bone grinding was performed to flatten the crest of the ridge under cooled sterile saline irrigation. **d** Osteotomy preparation with implant centres fixed at 7.5 mm, 6.5 mm, and 5.5 mm apart, so that four implants could be placed with inter-implant thread distances of 3 mm, 2 mm, or 1 mm. **e** Implants inserted. **f** Flap closure. **g** Osteotomy preparation to give implant thread to outer buccal bone distances of > 1 mm, 0.5–1 mm, and < 0.5 mm. **h** Resorbable barrier membrane placed over the implants and the buccal aspect of the ridge prior to flap closure

Two surgical interventions were performed in each of the 10 animals. First, the mandibular premolars (P2–P4) and first molar (M1) were extracted via a flapless approach. After a 12-week healing period [26], the alveolar ridge was exposed on both sides of the mandible following incision and reflection of the muco-periosteal flap. Gentle bone grinding was performed to flatten the crest of the ridge under cooled sterile saline irrigation (Fig. 1a–c).

#### Implant dimensions

The implants selected for the study were BLX® Roxolid® SLActive® 4.5 mm × 8 mm implants (The Straumann Group, Basel, Switzerland) that had a body diameter of 3.5 mm at the shoulder tapering to 1.9 mm at the apex. The cutting threads commenced 1 mm apical to the shoulder and terminated at the apex. The outer diameter of the peaks of the cutting threads was 4.5 mm in the coronal portion of the implant gradually reducing to 3.6 mm at the apex. Thus, the depth of the thread was 0.5 mm near the implant shoulder, gradually increasing to 1.3 mm at the apex. The neck of the implant consisted of 0.5-mm region of microthreads and a 0.5 mm coronal unthreaded region adjacent to the shoulder.

#### Inter-implant distance

On one side of the mandible, osteotomies were prepared in the centre of the ridge using a manufactured drill template, with the positions of the implants marked with a needle drill (Ø 1.6 mm; The Straumann Group, Basel, Switzerland). The implant centers were fixed at 7.5 mm, 6.5 mm and 5.5 mm apart, so that four implants could be placed with inter-implant TP distances of 3 mm, 2 mm, or 1 mm (Fig. 1d–f). At these TP distances, the corresponding distances between the shoulder of the implants was 4 mm, 3 mm, and 2 mm respectively. The needle drill was left in place after the first drill hole so that the drill template remained in place for the positioning of the other three implants. Osteotomy creation followed the manufacturer’s guidelines for hard bone type (BLX drills Ø 2.8 mm, 3.2 mm, 3.7 mm, and 4.2 mm, The Straumann Group, Basel, Switzerland). Four BLX Roxolid® SLActive® 4.5 mm × 8 mm implants (The Straumann Group, Basel, Switzerland) were placed at crestal bone level according to the manufacturer’s instructions. Closure screws were placed and the soft tissue repositioned and closed with resorbable sutures.

#### Buccal bone thickness

On the left side of the mandible, three implants were placed with the distance from the outer buccal bone to the centre of each implant was measured with a periodontal probe with 0.5-mm increments (HuFriedy PPSG Goldstein Colorvue Probe, HuFriedyGroup, Chicago IL, USA); the proposed implant centre was marked with a needle drill (as for the inter-implant distance above) and distances of 2 mm, 3 mm and 4 mm were measured to give distances from implant shoulder to outer buccal bone of 0.25 mm, 1.25 mm, and 2.25 mm, and TP to outer buccal bone distances of < 0.5 mm, 0.5–1 mm, and > 1 mm respectively (Fig. 1g–i). The implants were placed with a minimum of 4 mm distance between implant threads. Osteotomy creation and implant placement followed the same procedure as for the inter-implant distance protocol above. Due to the risk of microfractures of the buccal bone plate in the < 0.5 mm group, a slow resorbing collagen membrane (Straumann® Jason® membrane, Straumann AG, Basel, Switzerland) was placed over the buccal aspect of all implants on the left side to serve as a barrier membrane to protect the bone. No randomization was performed for either procedure.

**Fig. 2:**
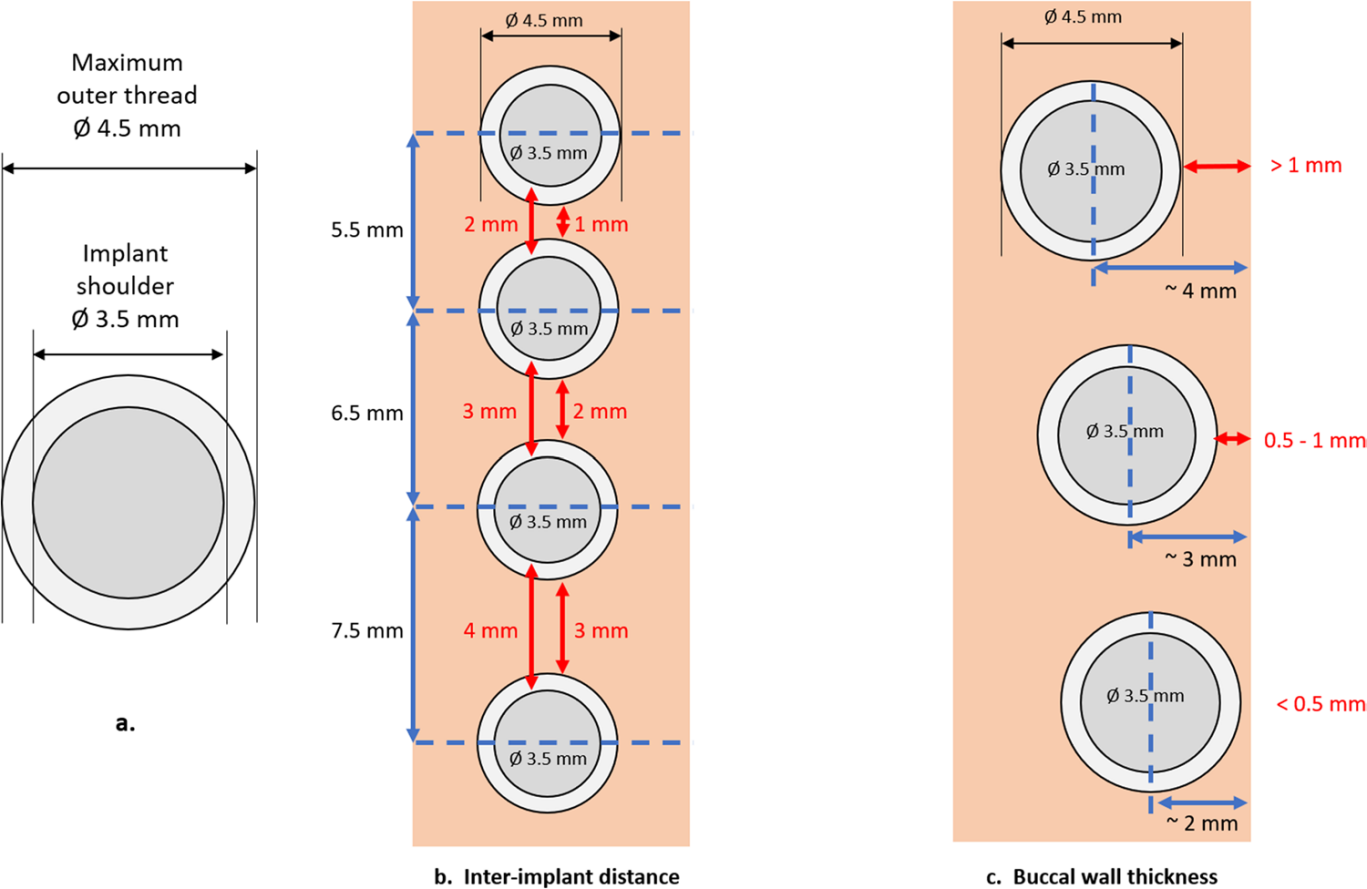
Schematic representation of **a** implant shoulder and maximum outer thread diameters in mm (Ø), **b** inter-implant distances of 1 mm, 2 mm, and 3 mm at maximum outer thread diameter, and **c** buccal bone thickness of < 0.5 mm, 0.5–1 mm, and > 1 mm from thread peak to outer buccal bone (not drawn to scale)

The relative dimensions of the implant and maximum outer thread diameter, as well as the inter-implant distances and buccal bone thickness are depicted in Fig. 2. All observations, including measured maximum insertion torque, as well as the inter-implant distance and buccal bone thickness measured by means of a periodontal probe, were recorded and photographs taken from all sites at implantation and termination.

### Post-surgery

The animals were observed until full recovery. They were housed in pens in groups of at least 3 animals under controlled environmental, and provided with a standard soft food diet for Minipigs. Twelve weeks after surgery all animals were sacrificed by intra-cardiac injection of a 20% solution of pentobarbital (Pentobarbitalnatrium, Apoteket AB; Stockholm, Sweden), 60 mg/ml.

### Sample preparation and processing

Block resections of the implant sites were prepared using an oscillating saw to preserve the soft tissue. The mandibles were fixed in formalin (formaldehyde 4% solution) for a minimum of 2 weeks prior to transporting for histological processing. Following immersion in formalin and dehydration via ascending grades of alcohol and xylene, the bone samples were infiltrated and embedded in methylmethacrylate for non-decalcified sectioning. Each hemi-mandible was separated into five blocks by bucco-lingual cuts through the center of the implant. The inter-implant distances were determined using the three middle blocks, each of which contained half an implant and the inter-implant region. Each block was again cut in a mesio-distal direction through the centre of the implant. For the calculation of buccal bone thickness, each implant was also was cut through the implant centre in the bucco-lingual direction. For both inter-implant and buccal bone thickness calculations, 500-µm-thick sections were obtained and ground to 30–50 µm, then stained with Paragon (toluidine blue and basic fuchsin) for microscopic evaluation.

### Histomorphometrical analysis

**Fig. 3:**
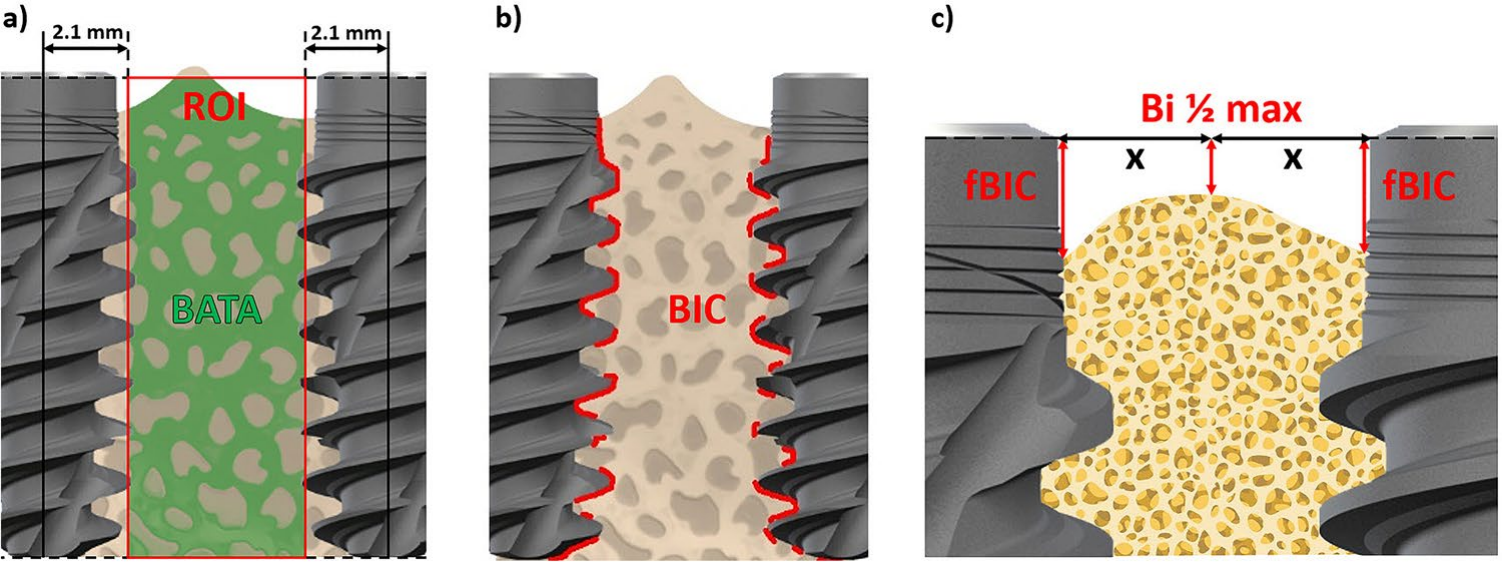
Histomorphometrical evaluations for inter-implant distance: **a** region of interest (ROI) for bone area to total area (BATA); **b** region measured for total bone-to-implant contact (BIC); **c** first bone-to-implant contact (fBIC) and interproximal level at the mid-distance between two implants (Bi ½ max)

From the stained sections obtained the following parameters were measured and calculated:

For inter-implant distance measurements:

- Bone area to total area (BATA, Fig. 3a), calculated as the percentage of bone tissue within the region of interest (ROI) between implants. This measurement was standardized between the different groups by drawing a line between the adjacent implant shoulders and between the adjacent implant apices since only the residual inter-implant bone area between two adjacent implants is described.
- Bone to implant contact (BIC, Fig. 3b), calculated as the percentage of implant surface in direct contact with bone. For each inter-implant region BIC was measured individually and then averaged.
- First bone to Implant contact (fBIC, Fig. 3c), calculated as the corono-apical distance between the implant shoulder and the most coronal contact point between bone and implant surface. For each inter-implant region, the fBIC was measured individually and then averaged. Negative values indicate a position apical to the implant shoulder.
- Coronal bone height at the mid-distance between two implants (Bi ½ max, Fig. 3c), measured parallel to the implant axes from a reference line drawn between adjacent implant shoulders.

For buccal bone thickness measurements:

- fBIC was measured as for the inter-implant distance, but only the buccal side was measured.
- BIC was measured as for the inter-implant distance, but only the buccal side was measured.
- Perpendicular crest to implant shoulder (pCIS), measured as the corono-apical distance between the implant shoulder and the most coronal bone (perpendicular to the implant axis), was used to evaluate buccal MBL changes. Using the knowledge that the stain utilised penetrates more easily into newly formed bone than into older residual bone, the two can be differentiated and therefore be used to measure two different pCIS values i.e. old versus new bone. This was considered as the coronal bone remodelling during the healing process and described as Delta pCIS.

### Statistical analysis

Descriptive statistics (means, SDs, medians, and ranges) were calculated, and paired comparisons were performed using the Wilcoxon signed rank test. Associations with each of the outcomes, adjusted for the effects of the animals, were examined using multivariable mixed linear regression models with adjustment for different group effects and multiple comparisons by the Dunnett-Hsu method.

## Results

**Fig. 4:**
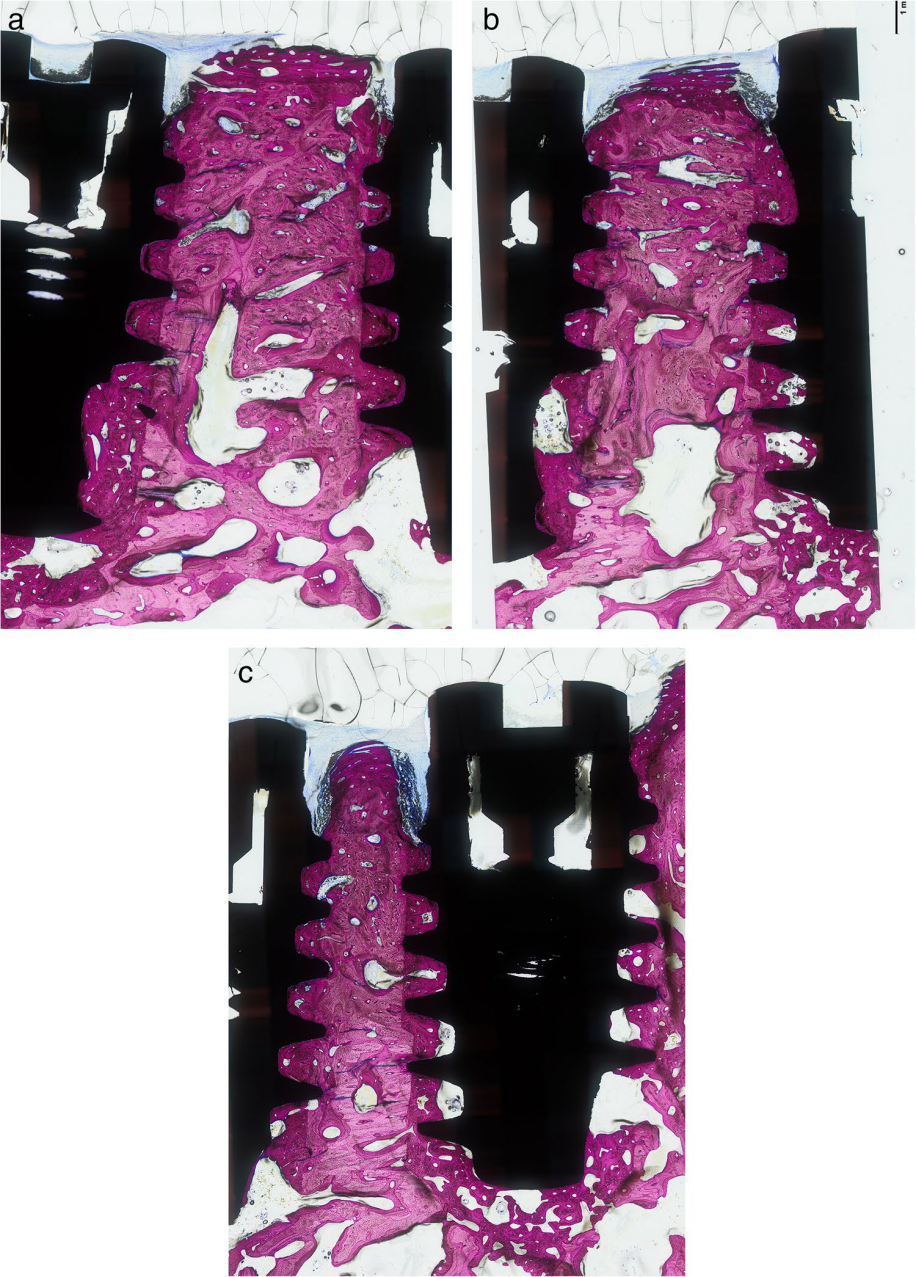
Histologic sections of inter-implant distances of **a** 3 mm, **b** 2 mm, and **c** 1 mm

**Fig. 5:**
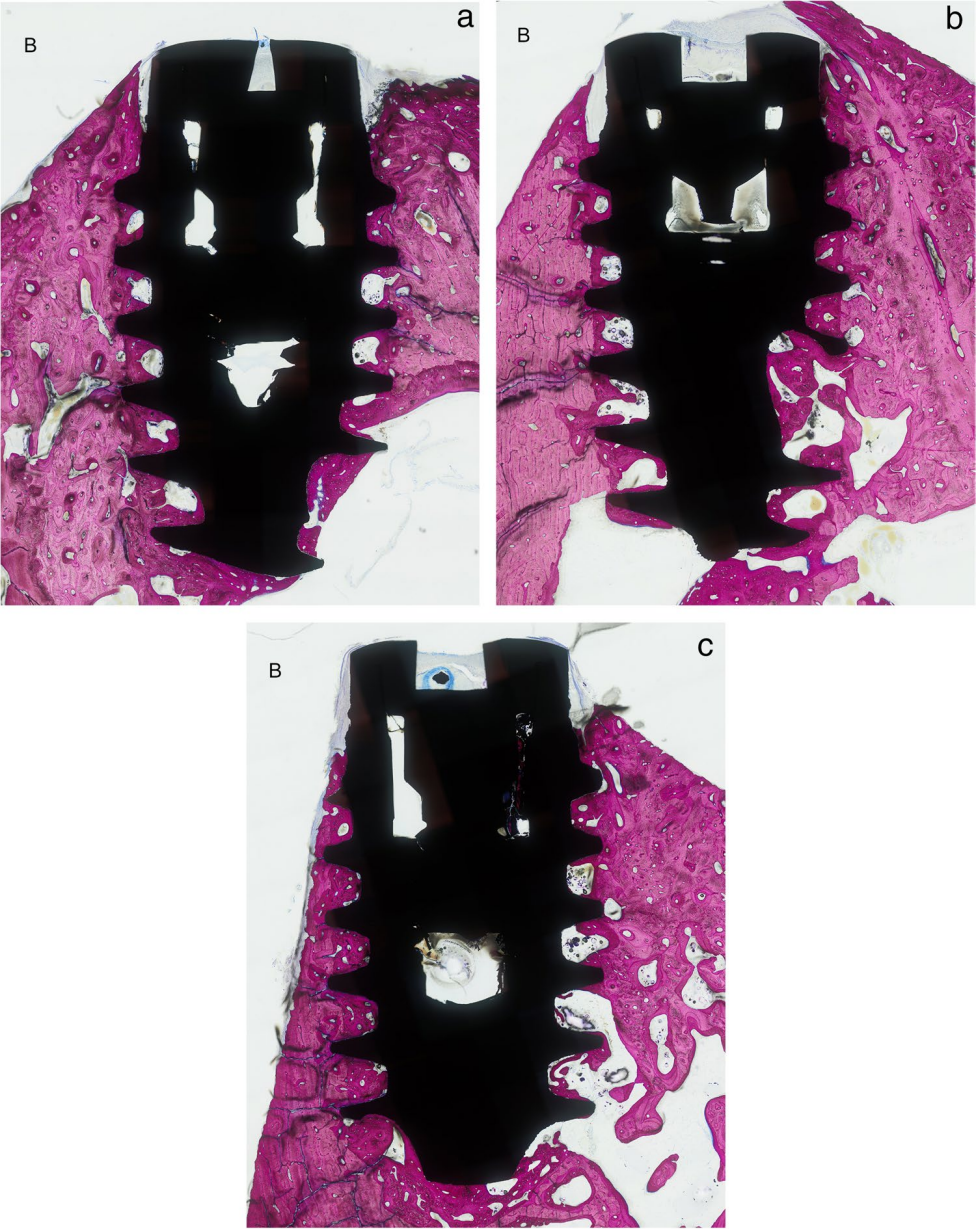
Histologic sections of buccal bone distances of **a** > 1 mm, **b** 0.5–1 mm, and **c** < 0.5 mm (buccal side denoted by letter B)

Recovery from surgery was uneventful in all animals. Of the ten animals implanted, one was used to test the surgical procedure and was therefore excluded from the subsequent analysis. The results from the remaining nine animals were therefore used for the analysis for inter-implant distances (Fig. 4a–c) and buccal bone thickness (Fig. 5a–c). There were no differences between groups for maximum insertion torque which ranged from 50 to 80 Ncm.

### Inter-implant distance

Drill templates were used to ensure correct implant positioning for inter-implant TP distances of 1 mm, 2 mm, and 3 mm; however, histological measurement showed slight variations and were recorded as follows:

- 3 mm group: mean TP distance 3.12 ± 0.39 mm (9 histology slides)
- 2 mm group: mean TP distance 2.11 ± 0.26 mm (9 histology slides)
- 1 mm group: mean TP distance 1.21 ± 0.20 mm (9 histology slides)

**Fig. 6:**
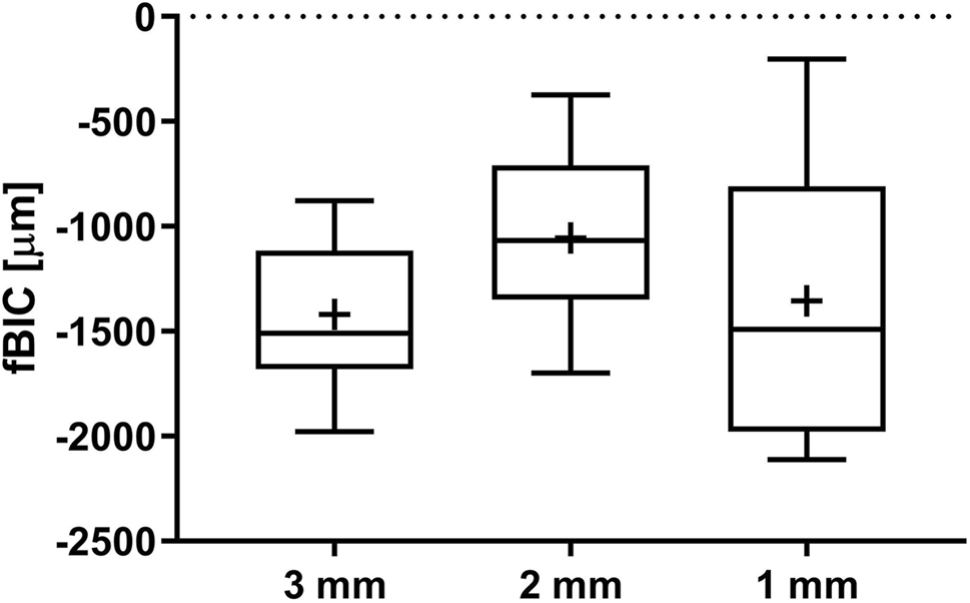
Median (horizontal lines), mean ( +), 25th and 75th percentile (boxes) values for first bone-to-implant contact (fBIC) at adjacent implants; whiskers show min. and max. values

**Fig. 7:**
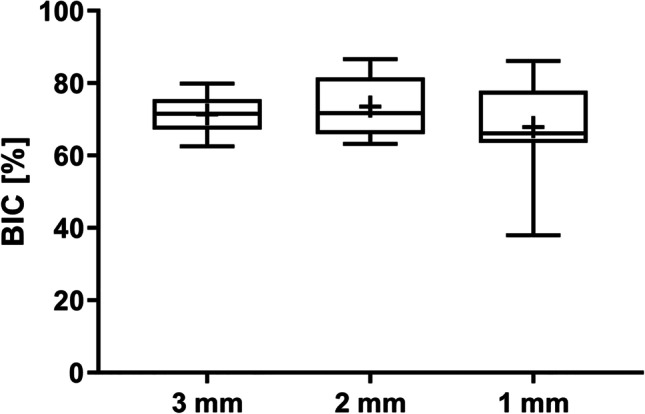
Median (horizontal lines), mean ( +), 25th and 75th percentile (boxes) values for total bone-to-implant contact (BIC) at adjacent implants; whiskers show min. and max. values

The mean fBIC for each slide was calculated from the mesial and distal fBIC measurements. The lowest mean fBIC was found in the 3 mm group (mean − 1420.33 ± 349.24 µm), followed by the 1 mm group (mean − 1356.18 ± 671.53 µm) and the 2 mm group (mean − 1054.71 ± 411.66 µm). No significant differences were observed between the groups. Figure 6 shows the median, 25th and 75 percentile values, showing that variation was greater in the 1 mm group, despite similar means. Wilcoxon signed rank test *p*-value showed no significant differences between the groups. Likewise, comparisons of the values adjusted for factor ‘animal’ using a mixed linear regression model (Table 1) also show no significant differences between the groups for fBIC. The *p*-value for the overall effect (*p* = 0.2052) was also not significant, indicating that inter-implant TP distance has no effect on the fBIC. Percentage of total BIC was comparable between the groups. The lowest value was in the 1 mm group (mean 67.91 ± 13.77%), followed by the 3 mm group (mean 71.37 ± 5.53%) and the 2 mm group (mean 73.55 ± 8.64%). As with fBIC, greater variation was noted in the 1 mm group (Fig. 7). Neither Wilcoxon signed rank test *p*-values nor the mixed linear regression model for adjusted associations (Table 1) showed any significant differences between the groups. The* p*-value for the overall effect (*p* = 0.5924) demonstrated that inter-implant TP distance had no effect on the total BIC.

**Table 1:** Adjusted outcomes for inter-implant distance measurements using mixed linear regression models

Outcome	Factor	Value	Regression parameters			Adjusted^§^ parameters for multiple comparisons				*p*-value (for the overall effect of the factor)
			Estimate	SE	*p*-value (*t*-test)_Reg_	Adjusted^§^ mean	95% CI for the adjusted mean	Dunnett-Hsu^†^ *p*-value		
Bi 1/2 max [µm]	Intercept	–	94.76	152.51	0.5517					
	Inter-implant distance	1 mm	− 142.70	175.62	0.4284	− 47.94	− 376.50–280.63	***Ref***	0.6422	0.5031
		2 mm	70.93	175.62	0.6917	165.69	− 162.87–494.25	0.4117	0.8919	
		3 mm	0			94.76	− 228.55–418.07	0.6351	***Ref***	
First bone to implant contact [µm]	Intercept	–	− 1420.33	166.02	< .0001					
	Inter-implant distance	1 mm	63.31	206.71	0.7634	− 1357.02	− 1714.12– − 999.91	***Ref***	0.9352	0.2052
		2 mm	366.45	206.71	0.0953	− 1053.88	− 1410.98– − 696.77	0.2870	0.1664	
		3 mm	0			− 1420.33	− 1772.28– − 1068.37	0.9336	***Ref***	
Bone to impact contact [%]	Intercept	–	71.37	3.32	< .0001					
	Inter-implant distance	1 mm	− 2.74	3.98	0.5014	68.63	61.49–75.77	***Ref***	0.7235	0.5924
		2 mm	1.47	3.98	0.7171	72.83	65.70–79.97	0.4999	0.9081	
		3 mm	0			71.37	64.34–78.39	0.7178	***Ref***	
BATA inter-implant [%]	Intercept	–	74.98	2.45	< .0001					
	Inter–implant distance	1 mm	− 2.40	3.01	0.4366	72.58	67.31–77.85	***Ref***	0.6510	0.2358
		2 mm	3.099	3.01	0.3186	78.08	72.81–83.35	0.1636	0.5022	
		3 mm	0			74.98	69.79–80.17	0.6452	***Ref***	

^§^The factor animal was introduced in the model as a random effect in a mixed linear regression model

^†^Adjusted for multiple comparisons

*Ref.* Reference level for the comparison within a factor; *BATA* bone area to total area, *pCIS* perpendicular crest to implant shoulder

**Fig. 8:**
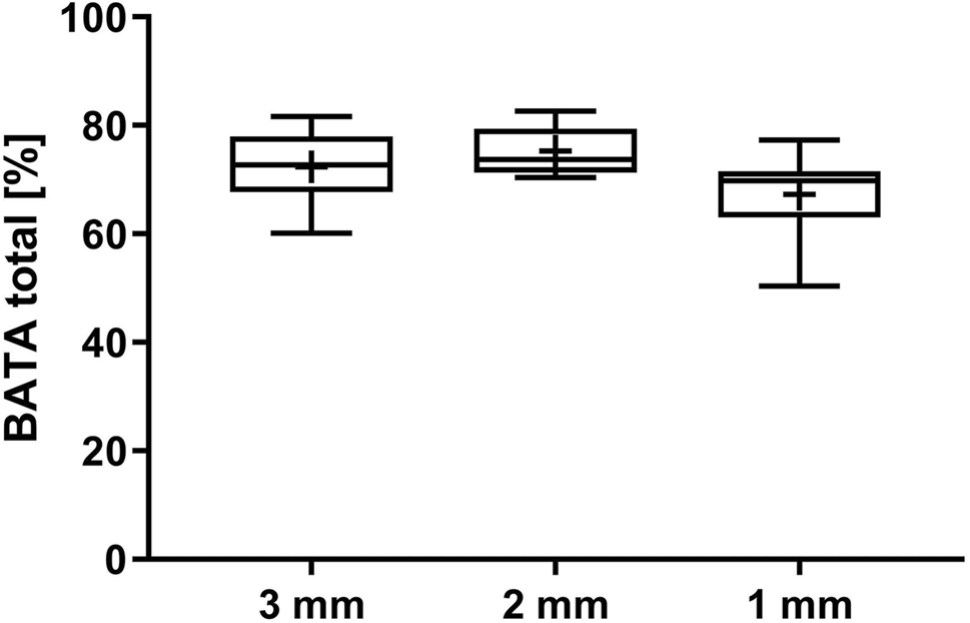
Median (horizontal lines), mean ( +), 25th and 75 percentile (boxes) values for bone area to total area (BATA) at adjacent implants; whiskers show min. and max. values

BATA was also comparable between the groups, with mean values of 72.06 ± 8.10%, 78.61 ± 5.98%, and 74.98 ± 7.71% for the 1 mm, 2 mm, and 3 mm groups, respectively. Figure 8 shows the median, 25th and 75th percentile values. No significant differences were observed using the Wilcoxon signed rank test or with adjusted comparisons using the mixed linear regression model (Table 1), and the *p*-value for the overall effect (*p* = 0.2358) showed that inter-implant TP distance has no effect on inter-implant BATA.

**Fig. 9:**
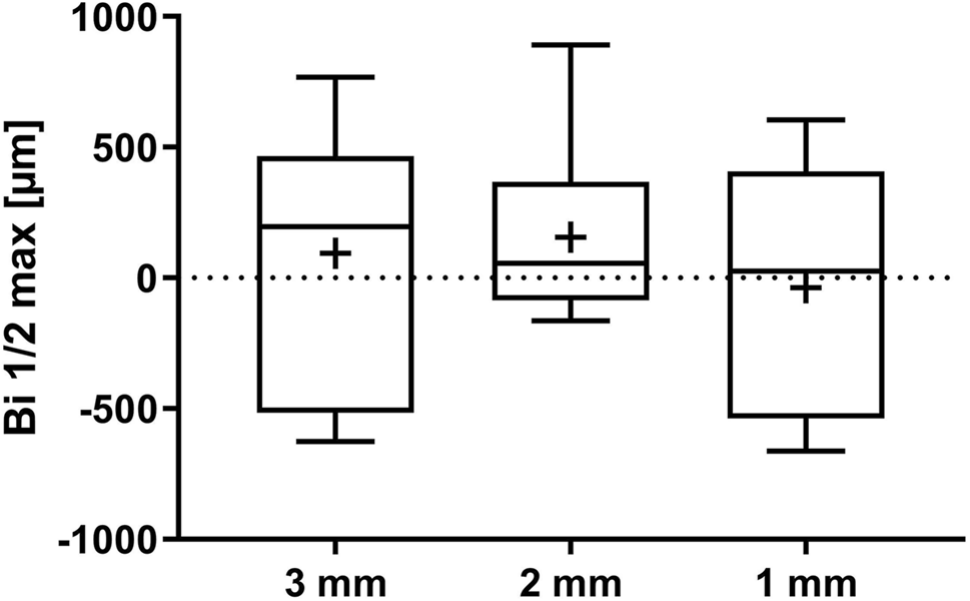
Median (horizontal lines), mean ( +), 25th and 75th percentile (boxes) values for coronal height at the mid-distance between two implants (Bi ½ max); whiskers show min. and max. values. The dotted line represents the crestal bone level at implant placement

Mean Bi ½ max values were − 38.31 ± 483.78 µm, 156.06 ± 355.94 µm, and 94.76 ± 515.12 µm for the 1 mm, 2 mm, and 3 mm groups, respectively. Median, 25th and 75th percentile values are shown in Fig. 9. Differences between the groups were not significant using the Wilcoxon signed rank test or the mixed linear regression model (Table 1). The *p*-value for the overall effect (*p* = 0.5031) indicated that inter-implant TP distance has no effect on Bi ½ max.

### Buccal bone thickness

The distance from the implant shoulder to the outer buccal bone was confirmed at implant placement using a periodontal probe to the closest 0.5 mm, where the implant centre was marked with a needle drill based on freehand measurements with the probe. The buccal bone thickness measured as the distance from implant shoulder to outer buccal bone was 0.25 ± 0.27 mm (range 0 to 0.5 mm), 1.0 ± 0.26 mm (range 0.5 to 1 mm), and 1.93 ± 0.18 mm (range 1.5 to 2.0 mm) for the < 0.5 mm, 0.5–1.0 mm, and > 1 mm groups respectively. The mean histological distance from TP to outer buccal bone by group was as follows:

-  < 0.5 mm group: mean distance 0.08 ± 0.13 mm; range 0–0.25 mm (9 histology slides)
- 0.5–1.0 mm group: mean distance 0.75 ± 0.13 mm; range 0.5–1.0 mm (8 histology slides)
-  > 1 mm group: mean distance 1.75 ± 0.22 mm; range 1.25 to 2.0 mm (10 histology slides)

**Fig. 10:**
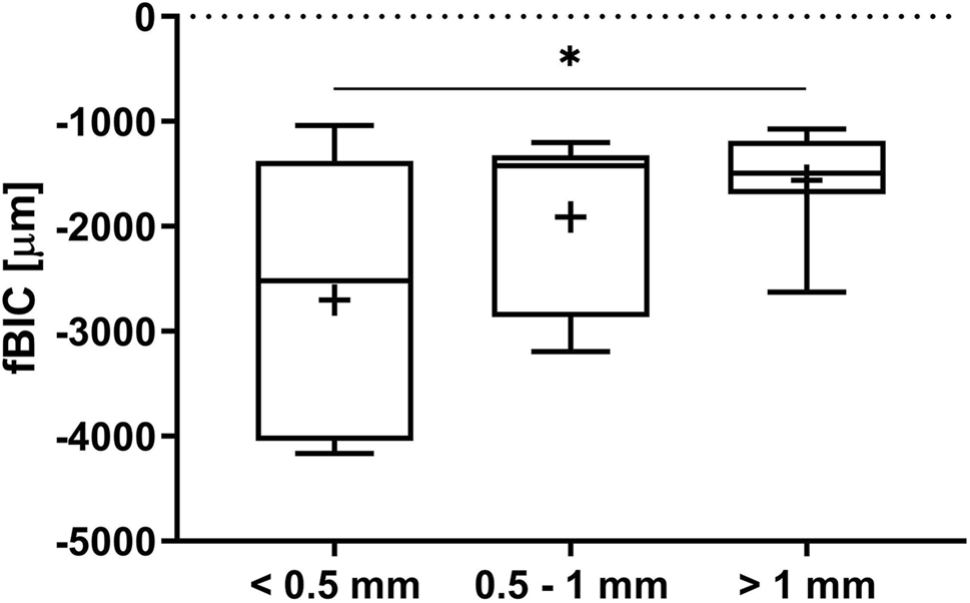
Median (horizontal lines), mean ( +), 25^th^ and 75 percentile (boxes) values for first bone-to-implant contact (fBIC) for implants at buccal bone sites; whiskers show min. and max. values. The dotted line represents the crestal bone level at implant placement

**Fig. 11:**
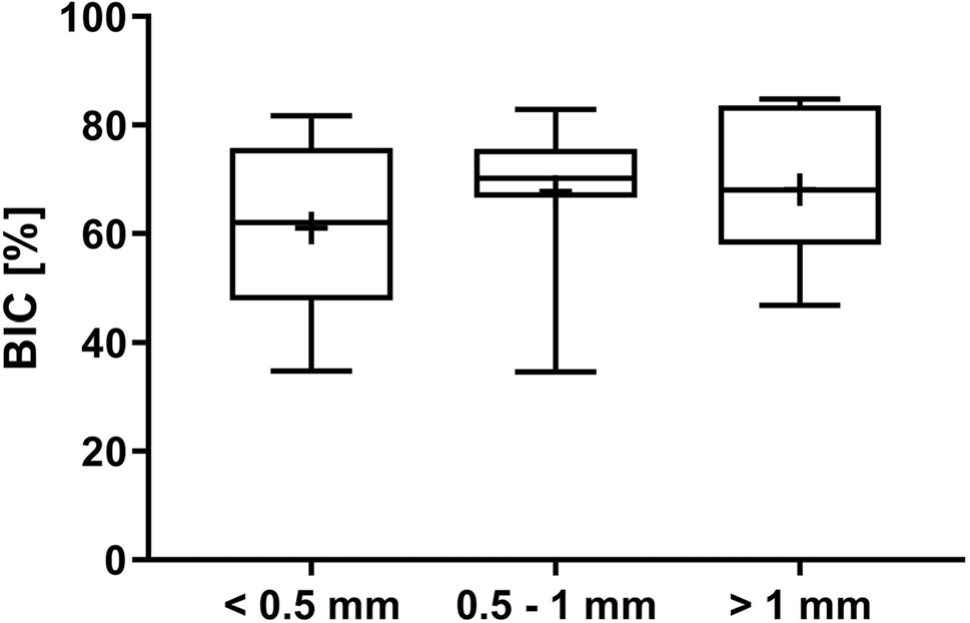
Median (horizontal lines), mean ( +), 25th and 75th percentile (boxes) values for total bone-to-implant contact (BIC) for implants at buccal b sites; whiskers show min. and max. values

Values for fBIC decreased as the buccal bone thickness increased. The lowest fBIC was observed in the < 0.5 mm group (− 2703.08 ± 1244.92 µm), followed by the 0.5–1 mm group (− 1911.99 ± 823.32 µm) and the > 1 mm group (− 1562.18 ± 464.08 µm). The < 0.5 mm group showed the greatest variation in values (Fig. 10). The Wilcoxon signed rank test showed borderline significance between the < 0.5 mm and the > 1 mm groups (*p* = 0.0547; Table 2), while the mixed linear regression model showed a significant difference for fBIC between the < 0.5 mm and the > 1 mm groups (*p* = 0.0179). The *p*-value for the overall effect was 0.0286, indicating that buccal bone thickness has a significant effect on fBIC. The same trend was not observed for total BIC, which showed similar values between the groups: mean percentage BIC was 61.00 ± 15.92%, 67.81 ± 14.40%, and 68.08 ± 14.13% for the < 0.5 mm, 0.5–1 mm, and > 1 mm groups, respectively. Figure 11 shows the median, 25th and 75th percentile values. No significant differences between the groups were observed using the Wilcoxon signed rank test or the mixed linear regression model, and the *p*-value of 0.5295 for the overall effect indicated that buccal bone thickness had no effect on total BIC.

**Table 2:** Adjusted outcomes for buccal bone thickness measurements using mixed linear regression models

Outcome	Factor	Value	Regression parameters			Adjusted^§^ parameters for multiple comparisons				*p*-value (for the overall effect of the factor)
			Estimate	SE	*p*-value (*t*-test)_Reg_	Adjusted^§^ mean	95% CI for the adjusted mean	Dunnett-Hsu^†^ *p*-value		
First bone to implant contact [µm]	Intercept	-	− 1563.47	284.75	0.0006					
	Bone thickness	< 0.5 mm	− 1139.61	387.85	0.0096	− 2703.08	− 3333.37– − 2072.78	***Ref***	0.0181	0.0286
		0.5–1 m	− 346.90	403.24	0.4023	− 1910.38	− 2577.89– − 1242.85	0.1256	0.6109	
		> 1 mm	0			− 1563.47	− 2167.11– − 959.83	0.0179	***Ref***	
Bone to implant contact [%]	Intercept	–	68.08	4.6882	< .0001					
		< 0.5 mm	− 7.08	6.8118	0.3139	61.0011	50.53–71.48	***Ref***	0.4971	0.5295
		0.5–1 m	− 0.28	7.0323	0.9688	67.8050	56.69–78.92	0.5519	0.9989	
		> 1 mm	0			68.0840	58.15–78.02	0.4923	***Ref***	
New pCIS [µm]	Intercept	–	− 455.26	251.26	0.1076					
		< 0.5 mm	− 941.92	365.08	0.0201	− 1397.19	− 1958.65– − 835.72	***Ref***	0.0372	0.0385
		0.5–1 m	− 52.45	376.89	0.8911	− 507.72	− 1103.24–87.81	0.0630	0.9862	
		> 1 mm	0			− 455.26	− 987.92–77.39	0.0368	***Ref***	
Old pCIS [µm]	Intercept	–	− 1255.25	208.35	0.0003					
		< 0.5 mm	− 2020.46	294.54	< .0001	− 3275.71	− 3739.23– − 2812.18	***Ref***	< 0.0001	< 0.0001
		0.5–1 m	− 181.75	305.11	0.5597	− 1437.01	− 1928.49– − 945.52	< .0001	0.7828	
		> 1 mm	0			− 1255.25	− 1696.92– − 813.58	< .0001	***Ref***	
Delta pCIS [µm]	Intercept	–	758.70	238.77	0.0130					
		< 0.5 mm	1119.827	296.20	0.0016	1878.52	1355.08–2401.96	***Ref***	0.0031	0.0043
		0.5–1 m	222.271	309.87	0.4837	980.91	429.40–1532.41	0.0210	0.7047	
		> 1 mm	0			758.70	252.53–1264.87	0.0031	***Ref***	

^§^The factor animal was introduced in the model as a random effect

^†^Adjusted for multiple comparisons

*Ref.* Reference level for the comparison within a factor, *pCIS* perpendicular crest to implant shoulder

Significant differences between groups were found for MBL, as measured by pCIS; new bone growth was discriminated from residual bone by measuring new pCIS and old pCIS, and the difference between the two was described as delta pCIS. Values for new pCIS decreased as the buccal bone thickness increased, indicating better MBL maintenance with increased bone thickness. There was a significant difference between the 0.5–1 mm group (mean − 507.72 ± 489.20 µm) and the < 0.5 mm group (mean − 1397.18 ± 1204.65 µm) by Wilcoxon signed rank test (*p* = 0.0391), while the mixed linear regression model showed no significant difference between these groups (*p* = 0.0630; Table 2). The difference between the > 1 mm group (mean − 455.26 ± 455.49 µm) and the < 0.5 mm group was borderline significant by Wilcoxon signed rank test (*p* = 0.0547) and significant by mixed linear regression (*p* = 0.0368; Table 2). Values for old pCIS also decreased as the buccal bone thickness increased. This indicated that initial marginal bone resorption following surgery and implant placement increased as buccal bone thickness decreased. The < 0.5 mm group (mean − 3275.71 ± 1051.12 µm) was significantly different from both the 0.5–1 mm group (mean − 1434.98 ± 264.19 µm) and the > 1 mm group (mean − 1256.88 ± 332.64 µm) by both the Wilcoxon signed rank test (*p* = 0.0156 and *p* = 0.0039, respectively) and the mixed linear regression model (p < 0.0001 for both differences; Table 2). The same pattern was observed for delta pCIS, i.e., the < 0.5 mm group (mean 1878.52 ± 1041.64 µm) was significantly different from the 0.5–1 mm group (mean 927.265 ± 515.75 µm) and the > 1 mm group (mean 801.61 ± 534.51 µm) by Wilcoxon signed rank test (*p* = 0.0234 and *p* = 0.0195, respectively) and mixed linear regression model (*p* = 0.0210 and *p* = 0.0031, respectively; Table 2). The *p*-values for the overall effects for new pCIS (*p* = 0.0385), old pCIS (*p* < 0.0001), and delta pCIS (*p* = 0.0043) indicated that buccal bone thickness has a significant effect on maintenance of buccal MBL.

## Discussion

This study aimed to determine whether placing adjacent implants of a novel design at varying inter-implant TP distances can have an influence on bone healing and inter-implant bone height. The secondary objective was to assess whether the implants can have an effect on maintenance of MBL if placed with TP closer to the outer edge of the buccal bone than the recommended 1 to 2 mm minimum [19]. The results indicated that inter-implant TP distance has no significant effect on bone healing when the implants are placed at TP distances of 3 mm, 2 mm, and 1 mm between adjacent implants, which corresponds to the distances between the shoulder of the implants of 4 mm, 3 mm, and 2 mm respectively. The 1 mm group had TP distance of 1 mm and an inter-implant shoulder distance of 2 mm, which were less than the original 3 mm recommendation of Tarnow and co-workers [13]. The authors based their recommendation on implants designed with an external hexagon connection in which an average MBL of 1.2 mm could be anticipated in the first year of function [27] accompanied by a V-shaped pattern of bone resorption at the neck of the implant, also referred to as crater- [28] or saucer-shaped defects [29]. If considering the implant shoulder as the reference for inter-implant distance, the findings of the present study are consistent with studies of contemporary implants which incorporate internal tapered abutment connections (ITAC) that offset the microgap at the implant-abutment interface from the implant shoulder. Implants with this design have been shown to have significantly less MBL than implants that did not offset the implant-abutment junction from the implant shoulder [30], and had less tendency to form a crater-shaped marginal defect [26]. Preclinical studies have shown that these implants can be placed 2 mm apart without compromising histologic and radiographic inter-implant MBL in a minipig model [31, 32] as well as in a canine model [33–36]. Recent clinical studies have confirmed the preclinical reports. For example, Koutouzis and colleagues evaluated 30 patients who had adjacent internal tapered abutment connection implants placed either 2 mm, 3 mm, or > 4 mm apart [37]. No differences in radiographic marginal or mid-proximal bone loss were seen in any of the groups at up to 2 years follow-up. However, unlike the novel implant used in the present study, the TP of these implants did not extend beyond the widest diameter of the implant body. In other words, the shoulder and threads of these implants were at least 2 mm apart. The findings of the present study are therefore unique, as this is the first report indicating that bone healing may not be adversely affected at an inter-implant shoulder distance of 2 mm and TP distance of 1 mm. In this regard, it is interesting to speculate whether the major factor influencing bone healing with this novel implant is the inter-implant TP distance or the inter-implant shoulder distance, and whether smaller inter-implant TP distances could influence healing outcomes. It was noted in the present study that the results for the 1 mm group did show greater variability for fBIC and %BIC suggesting that TP proximity of 1 mm may influence these parameters. A scanning electron microscope study of loaded dental implants in a canine model showed the presence of an intricate vascular network adjacent to the implant surface that was located within bone canals 10 to 70 µm in diameter. This vascular network was connected to the adjacent marrow tissues [38]. A study in a canine model found that the vascularity of the bone between implants was significantly less for inter-implant distance of 2 mm compared to 3 mm [39]. It is plausible, therefore, that the reduced marrow volume associated with TP proximity of 1 mm in the present study may adversely influence the vascularity of the inter-implant bone, and explain the variability in fBIC and %BIC observed in the 1 mm group. Further studies with the same implant body diameter but wider thread diameters and smaller inter-implant TP distances would be needed to clarify this.

For buccal bone thickness, the results of the present study showed that fBIC was significantly closer to the implant shoulder in the > 1 mm group compared to the < 0.5 mm group. For the > 1 mm group, the mean buccal bone thickness at the implant shoulder was 1.93 ± 0.18 mm and mean TP to outer bone distance was 1.75 ± 0.22 mm. This suggests that for this novel implant, a minimum TP to outer buccal bone thickness of 1.75 mm is required to minimize the distance fBIC to the implant shoulder.

The results also showed that MBL was similar for the 0.5–1.0 mm and > 1 mm groups, with both groups showing significantly better maintenance of MBL compared to the < 0.5 mm group. For the 0.5–1.0 mm group, the buccal bone width was 1 mm at the implant shoulder and the mean TP to outer buccal bone distance was 0.75 mm. The findings are interesting, as it suggests that under the conditions of this study, implant threads that are wider than the body diameter of the implant may not have a detrimental effect on buccal bone height maintenance, provided the thread tips are at least 0.75 mm and the buccal bone width at the implant shoulder is 1.0 mm. These findings are at odds with published clinical and preclinical studies which suggest that greater bone thickness of 1.5 mm to 2 mm is needed to maintain bone height. In a recently published pre-clinical study, implants were placed in canine mandibles in either thick (≥ 1.5 mm) or thin (< 1.5 mm) buccal bone. There was significantly greater peri-implant bone loss with a thinner buccal bone [40]. In a large prospective clinical study, 2685 implants that were placed in patients at 30 centres were evaluated [15]. It was found that implants with a buccal bone thickness of 1.8 to 2 mm at the time of placement had less corono-apical crestal bone loss at the surgical uncovering 6 months later than implants with buccal bone less than 1.8 mm in width. Indirect evidence for minimum buccal bone thickness may also be derived from clinical studies reporting on peri-implant soft tissue stability. In a recent clinical study, sites with initially ≥ 1.5 mm buccal bone thickness demonstrated coronal growth of soft tissues, compared to sites with < 1.5 mm of buccal bone thickness which recorded 0.64 to 1.22 mm of mucosal recession 3 years after implant placement [40, 41]. The reasons for the difference between the published literature and the present study in relation to the minimum buccal bone thickness are unclear, but may relate to differences in the study design (clinical vs. preclinical) and the preclinical models used (canine vs porcine), the limitation in manual measurements of intraoperative buccal bone thickness, or may be attributable to the novel design of the implant itself. Comparative studies would be required to shed further light in this. This study was designed with a submerged healing protocol. A transmucosal comparison was deemed unnecessary due to the results of a previous study using the same implant design and the same pre-clinical model which demonstrated no difference in the bone response between submerged and transmucosal healing [20].

As with all experimental studies, the authors note certain limitations in this study. In terms of study design, the aim was to perform an effective evaluation of the test situation ethically, using as few animals as possible, this does mean that there was (a) no randomization, since the implant positioning was exactly the same in each animal, and (b) no control group using a more standard implant design, i.e., one where the implant shoulder is the widest part of the implant. In addition, while the results of this study suggest that the BLX implant can be placed with the TP 1 mm apart and as close as 0.5 to 1 mm to the outer surface of the buccal bone, these findings may not be directly translatable to the clinical setting and should be interpreted cautiously. In particular, implants placed too close together may have negative consequences in terms of access for maintenance and homecare in a clinical setting. Clinical studies designed to evaluate these parameters are required.

## Conclusions

The results from this preclinical study suggest that the inter-implant bone levels between adjacent BLX implants can be maintained even when the distance between TP is as low as 1 mm. A minimum TP to outer buccal bone distance of 0.75 mm, which corresponds to buccal bone thickness of 1 mm at the implant shoulder, is required for predictable maintenance of buccal MBL. However, fBIC distance to the implant shoulder was lowest with TP to outer buccal bone distance of 1.75 mm corresponding to buccal bone thickness of 2 mm at the implant shoulder.
